# Assessment of quality of care provided to adults with type 2 diabetes mellitus at public hospitals in Gamo Gofa zone, Southern Ethiopia: Facility based Cross‐Sectional study

**DOI:** 10.1002/edm2.355

**Published:** 2022-06-27

**Authors:** Teklu Teshome Russo, Mende Mensa Sorato, Akililu Ayele Mesfin, Tadiwos Hailu, Abayneh Tunje Tanga, Zebenay Bussa

**Affiliations:** ^1^ Department of Biomedical Sciences, College of Medicine and Health Sciences Arba Minch University Arba Minch Ethiopia; ^2^ Department of Pharmacy, College of Medicine and Health Sciences Arba Minch University Arba Minch Ethiopia; ^3^ School of Medicine, College of Medicine and Health Sciences Arba Minch University Arba Minch Ethiopia; ^4^ School of Public Health, College of Medicine and Health Sciences Arba Minch University Arba Minch Ethiopia

**Keywords:** quality of care, southern Ethiopia, theory is Donabedian triad model, type 2 diabetes mellitus

## Abstract

**Purpose:**

Proactive management of type 2 diabetes is important for restoring beta‐cell function and improving sustained blood glucose control. Evidence on quality of diabetes care in Ethiopia is inadequate.

**Method:**

Facility‐based cross‐sectional study was conducted to assess level of quality of care provided to adult type 2 diabetes patients at three public hospitals in Gamo Gofa Zone, Southern Ethiopia.

**Results:**

A total of 210 adult type 2 diabetes patients were included. The mean age of patients was 44.1 ± 9.94 years. Fifty‐one (24.3%) of patients adhered to prescribed medicines. Sixty‐seven (31.9%) patients could benefit from neuropathy screening and referral. Diabetes‐specific evidence‐based guidelines, operational plan to reduce overweight and obesity were not available. There was no periodic lipid profile, renal function and glycated haemoglobin testing. Sixty‐three (30%) patients achieved fasting blood glucose (FBG) level. Only 41 (19.5%) achieved the recommended target value for composite intermediate outcomes. All three sub‐components of quality care structure, process and outcome (SPO) were below the agreed minimum score and the quality of care provided to adult type 2 diabetes was poor. Only 41 (19.5%) achieved agreed quality indicator targets for type 2 diabetes (fasting blood glucose blood pressure and low‐density lipoprotein cholesterol).

**Conclusion:**

The quality of care provided to adult type 2 diabetes patients was poor particularly in areas such as availability of evidence‐based guidelines, operational plan to reduce obesity, monitoring of lipid profile and glycaemic control. Therefore, developing strategies for addressing structure, process and outcome‐related gaps by involving all stakeholders is critical for improving the quality of care provided to these patients.

## Graphical abstract

Only 41 (19.5%) achieved agreed quality indicator targets for type 2 diabetes (fasting blood glucose, and blood pressure, and low density lipoprotein cholesterol).The quality of care provided to adult type 2 diabetes patients was poor particularly in areas like availability of evidence based guidelines, operational plan to reduce obesity, monitoring of lipid profile and glycaemic control.

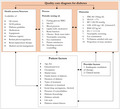

## 1 INTRODUCTION

Diabetes mellitus is a chronic metabolic disorder of multiple aetiologies caused by the failure of cells of the body to metabolize sugar properly due to a total or relative lack of insulin.^1^ Type 2 diabetes accounts for 90–95% of diabetes cause and it threatens the economies of all nations, particularly developing countries.^2^ Every 5 s, one person dies because of diabetes and its complications.^3^ Over 4 in 5 (81%) adults with diabetes live in low‐ and middle‐income countries (LMICs).^4^

Proactive management (metabolic surgery, intensive therapeutic interventions or significant lifestyle modification) of type two diabetes is important for restoring beta‐cell function and improving sustained blood glucose control.^5^ Controlling diabetes and its key risk factors are strongly reflected in the nine voluntary global targets to reach by 2025. These targets include^1^ a 25% reduction in overall mortality from cardiovascular disease, cancer, diabetes and chronic respiratory disease^7^; halt rise in obesity and diabetes^8^; at least 50% of eligible people receive drug therapy and counselling to prevent heart attack and stroke and^9^ at least 80% availability of affordable basic technologies and essential medicines in both public and private facilities.^6^

**FIGURE 1:**
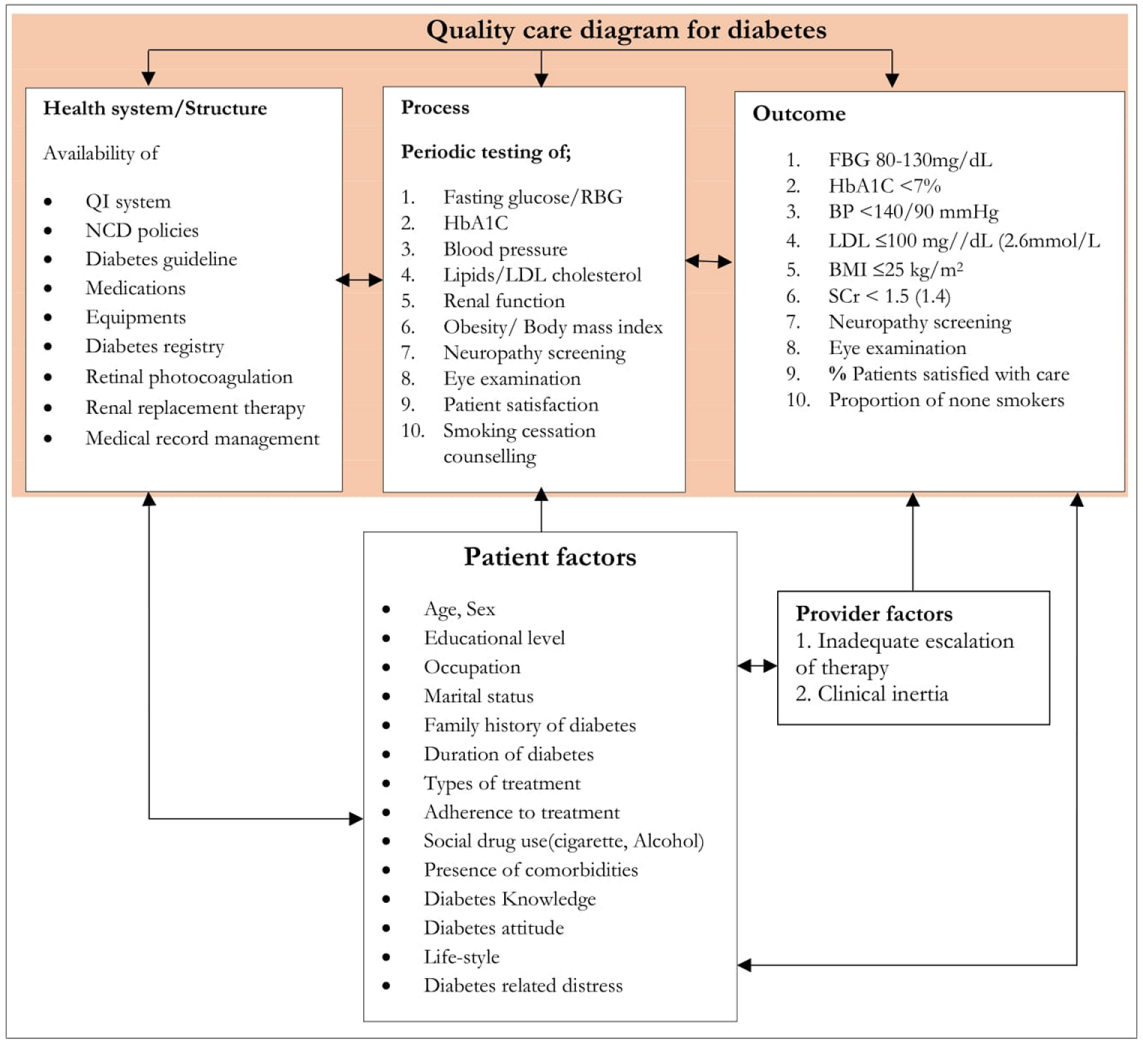
Conceptual framework for quality of diabetes care based on structure, process and outcome theory for developing countries. Adapted from donabedian, other diabetes treatment guidelines and different literatures

Quality of care is the application of medical science and technology in a way that maximizes its benefits to health without correspondingly increasing its risks.^7^ The most widely used healthcare quality assessment theory is Donabedian's Triad Model (Figure 1). Structure describes the material and human resources as well as the organizational structure. The structure/process quality indicators are aimed at internal quality assurance and they, therefore, enable comparisons between different programmes and also between healthcare providers conducting these activities. Outcome describes the effect of care or interventions on the health status of a subject or population.^8^, ^9^, ^10^

Based on the available data, no country consistently performs all indicators of quality of diabetes care, even those that spend much more on health.^11^, ^12^ The quality of diabetes care remains suboptimal worldwide regardless of the country's level of development or healthcare system.^12^ Similarly, diabetes and its care in Ethiopia have never been given the attention it deserves and glycaemic control and management of co‐morbid conditions and diabetes complications are alarmingly sub‐optimal.^13^ Very few studies on the factors influencing the quality care of patients with diabetes have been reported from LMICs,^14^, ^15^ despite over 81% of adults with diabetes living in these countries.^4^ To the best of our knowledge, none of the studies done have used a SPO model to assess the quality of diabetes care and associated factors in public hospitals in Southern Ethiopia. Therefore, this research was conducted to assess the level of quality of care provided to adults with type 2 diabetes at three public hospitals in Gamo and Gofa Zones, southern Ethiopia.

## 2 METHODS AND MATERIALS

### 2.1 Study design, area and period

A facility‐based cross‐sectional study will be conducted from (15 March to 15 May, 2020), at three public hospitals in Gamo and Gofa zones. The three hospitals namely Arba Minch General Hospital, Sawula General Hospital and Chencha District Hospital providing care for diabetes. According to the 2019 hospital health management information system (HMIS) report, there are about 547, 315 and 210 registered diabetes patients were receiving care from Arba Minch General Hospital, Sawula General Hospital and Chencha District, respectively.

### 2.2 Populations

The source population of this study was all adult type 2 diabetes patients who visited the selected public hospitals for follow‐up care and their respective charts. While the study population was those aged 18 years and older, diagnosed with type 2 diabetes and visited the selected hospitals at the time of the data collection period and those who fulfil the inclusion criteria.

### 2.3 Eligibility criteria

All adult type 2 diabetes patients (18 years of age and above) having at least 3‐month follow‐up before the time of data collection, patients who can give consent and patients' medical charts written clearly and complete were included. While patients with history of dementia, patients with hearing impairments or any other serious health problems, incomplete records and pregnant women were excluded.

### 2.4 Sample size determination and sampling technique

#### 2.4.1 Sample size determination

The sample size is determined by using single population proportion formula by taking a proportion of fasting blood glucose test done, which is one of the outcome indicators of quality of diabetes care as 85.6% from a study conducted in Jimma zone^16^ and *Z* value of 1.96 at 95% confidence interval will be used and 10% will be added for non‐response rate. After adding 10% for non‐response rate, 210 adult type diabetics will be included in this study.

$$n=\frac{{\left(\mathrm{Z\alpha}/2\right)}^{2}*P\left(1-P\right)}{d^{2}}=189.4$$

Where; *n* = is the sample size; *Zα*/2 = is the abscissa of the normal curve that cuts off an area α at the tails (1 ‐ *α* equals the desired confidence level, e.g., 95%) or standard normal deviation, set at 1.96, correspond to the 95% confidence interval; *d* = desired level of precision/margin of error; *p* = estimated proportion of fasting blood glucose measurement done (*p* = 85.6%), and *q* is 1–*p*.

#### 2.4.2 Sampling technique and procedures

Proportional allocation based on a number of adult type 2 diabetes patients on follow‐up at three hospitals was done. Ninety‐three patients from Arba Minch General Hospital, 67 patients from Sawula General Hospital and 50 patients from Chencha District Hospital were included in this study. A consecutive sampling technique until desired sample size is achieved was used to collect data. Patients coming to the chronic care clinic for a follow‐up service during data collection period were interviewed after screening them for eligibility criteria on arrival. Codes were given to the patient charts during the interview and coded charts were stored separately for later chart review to save patient time. The respective chart review was done after interviewing patients to collect most of the process and outcome indicators. Patients were interviewed in a separate room with their privacy. Both the interviews and chart reviews were done by registered professional nurses trained by investigators for data collection. Managers of respective hospitals, coordinators of chronic care unit and medical record officers were interviewed for data concerning structure‐related questions.

### 2.5 Variables of the study

#### 2.5.1 Independent variables

##### Patient‐related variables

Age sex, educational status, economic status, occupational, marital status, family history of diabetes, duration of diabetes, types of treatment, adherence to treatment, presence of comorbidities, lifestyle, diabetes‐related knowledge, attitude and diabetes‐related distress.

#### 2.5.2 Outcome variable

Quality of diabetes care (Structure‐Process‐Outcome).

### 2.6 Data collection tools procedures

For this study, the SPO indicators were used for evaluating the quality of care. The data were collected by using a structured questionnaire developed by the research team after reviewing similar studies conducted across the world and adapted to the country context.^17^, ^18^, ^19^, ^20^, ^21^, ^22^, ^23^, ^24^, ^25^, ^26^, ^27^ Patients were interviewed by trained data collectors about their demographic characteristics, disease‐related characteristics, diabetes‐related knowledge, attitude, medication adherence and diabetes‐related distress and their satisfaction with the care provided to them during the data collection period.

Medication adherence was evaluated by using, eight‐item Morisky Medication Adherence Scale (MMAS‐8), which is validated among the different populations for adults with diabetes. MMAS consists of 8 items with a dichotomous response (yes/no) for items 1–7 and a 5‐point Likert response for the last item. The total score ranges from 0 to 8 with a higher total score indicating higher medication adherence.^28^ Diabetes‐related knowledge and attitude tools were adapted from Michigan Diabetes Research Center tools for health professionals and adapted to the national context.^29^, ^30^, ^31^ Diabetes‐related distress of patients was assessed by using a validated tool containing 17 items adapted from a Pragmatic trial to reduce diabetes distress.^32^ Patient satisfaction about care received was assessed by interviewing patients using a short‐form patient satisfaction questionnaire (PSQ‐18), which is developed through rigorous research and abbreviated from much larger questionnaires, maintaining internal consistency and reliability and validated for use in different settings.^18^, ^22^, ^26^ We used the self‐administered Michigan Neuropathy Screening Instrument (MNSI) as a measure of distal symmetrical peripheral neuropathy among type 2 diabetes patients.^33^ Assessment of the presence of retinopathy screening service was done by reviewing the link of patients with ophthalmologist or optometrist service in the chart and patient interview.

Quality of care indicators was derived based on clinical practice guideline targets and the National Diabetes Quality Improvement Alliance (NDQIA) performance measures.^9^, ^34^, ^35^, ^36^ The diabetes quality care indicator contains a total of 28 items and is further divided into 3 sub‐divisions (structure, process and outcome), structure 8 items, process 10 items and outcome 10 items.

Structure‐related factors data were collected by interviewing the head of the facility, chronic care clinic and medical record officer. Availability of quality improvement and documentation system was rated as met and unmet for each criterion. Availability of technologies and guidelines was assessed by respective questionnaires adapted from the Ethiopian Hospital transformation assessment handbook.^21^ Major structure‐related variables that have a significant effect on type 2 diabetes and its complications (availability quality improvement system, availability of basic technologies for diabetes, general availability of diabetes medications and medical record management) were assigned to have 2 points weight each, while other variables (availability of policies documents, availability of diabetes registry, retinal photocoagulation and renal replacement therapy) were assigned to have 1 point weight each based on research team agreement. The total mean score for the structure is the sum of the responses to the appropriate items and divided by 12. We considered a mean item score of 0.6 or higher as a quality structure for providing care for adults with type 2 diabetes.

For process and outcome‐related variables, major variables that have a significant effect on type 2 diabetes and its complications (FBG/ HbA1C%, BP, LDL and BMI, smoking cessation) were assigned to have 2 points weight each, while other variables were assigned to have 1 point weight each based on research team agreement. The total mean score for the process is the sum of the patient's responses to the appropriate items and divided by 16. We considered a mean item score of 0.8 or higher as a quality process or outcome of care. Finally, the care is labelled as the quality of care if the three sub‐components were claimed as quality (i.e., quality structure, quality process and quality outcome) otherwise poor. Glycaemic control was defined by individual HbA1C% targets (≤7.0%) or mean FBG 80–130 mg/dl or RBS < 180 mg/dl.^37^, ^38^, ^39^

### 2.7 Data processing and analysis

#### 2.7.1 Data quality control

The patient interview part of the questionnaire was translated into Amharic and translated back into English to check its consistency. The Amharic version of the patient interview questionnaire and English version of data abstraction format and structure‐related questionnaires were used for data collection. The questionnaire was pretested on 20 adult diabetic patients in Arba Minch General Hospital to check for the consistency of questionnaire, and possible amendments were made based on findings. Seven professional nurses (BSc.) for data collection and one medical doctor (MD) working in the respective hospital for supervision were oriented before data collection about principles to follow during data collection and the contents of data collection format for 1 day by the principal investigators. Continuous follow‐up and supervision were done by the principal investigators throughout the data collection period.

#### 2.7.2 Data analysis

The collected data were checked for completeness and consistency by principal investigators on daily basis at the spot during the data collection time. Then, data were transcribed back to English for the patient interview part and entry was made using Epi‐data 3.1 software. After data processing, analysis was done by using SPSS version 20.0. A summary of descriptive statistics was computed for most variables such as socio‐demographic factors; structural factors, process and outcome indicators. A point estimates of Odds ratio (OR) with 95% confidence interval (CI) was determined to assess the strength of association. For all statistical significance, *p*‐value <.05 was used as a cut‐off point.

## 3 RESULTS

### 3.1 Socio‐demographic data

A total of 210 adult type 2 diabetes patients (51.9% females) were included in this study. Ninety (42.9%) were in the age range 41–50 years, with a mean age of patients was 44.1 ± 9.94 years ranging from 18 to 68 years. Concerning religion, the majority were Orthodox 91 (44.6%) followed by protestant 81 (39.7%). About level of education, 106 (50.5%) attended college and above followed by secondary school complete 39 (18.6%). Ninety‐five (45.2%) of patients were employed followed by merchants 51 (24.3%) (Table 1).

**TABLE 1:** Socio‐demographic characteristics of adult type 2 diabetics on follow‐up at public hospitals in Southern Ethiopia (*n* = 210)

Socio‐demographic characteristics		Frequency	Percent
Sex	Male	101	48.1
	Female	109	51.9
Age category	≤30 years	19	9.0
	30–40 years	53	25.2
	41–50 years	90	42.9
	51–60 years	35	16.7
	Above 60 years	13	6.2
Religion	Orthodox	91	44.6
	Protestant	81	39.7
	Muslim	35	16.7
	Catholic	3	1.4
Ethnicity	Gamo	93	44.3
	Gofa	85	40.5
	Wolayita	12	5.7
	Amhara	16	7.6
	Others	4	1.9
Marital status	Married	162	77.1
	Divorced	17	8.1
	Widowed	17	8.1
	Single	14	6.7
Level of education	Illiterate	36	17.1
	Primary school complete	29	13.8
	Secondary school complete	39	18.6
	College graduate and above	106	50.5
Occupation	Employed	95	45.2
	Merchant	51	24.3
	Unemployed	36	17.1
	Retired	20	9.5
	Others	8	3.8

### 3.2 Disease‐related characteristics data

**FIGURE 2:**
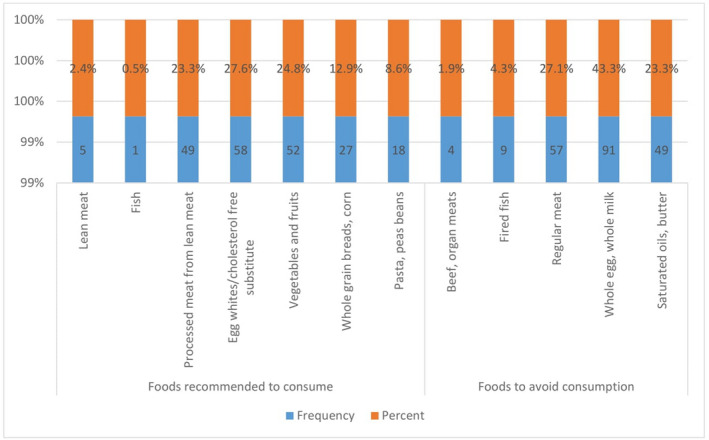
Patient reported foods to eat and foods to avoid for diabetes patients at among adult type 2 diabetics at Gamo and Gofa Zones

A mean duration of disease was 4.32 ± 2.39 years. A mean fasting blood glucose (FBG) was 145.55 ± 26.07 mg/dl ranging from 45 to 190 mg/dl. Sixty‐three (30%) of patients achieved their FBG target. Eighty‐seven (41.4%) patients were overweight with a mean body mass index (BMI) of 24.23 ± 3.27 kg/m^2^ ranging from 19 to 36 kg/m^2^. Concerning the type of medications used for the management, majority 179 (85.2%) were taking oral antidiabetic medications followed by insulin and oral antidiabetic combinations 31 (14.8%). Metformin was the most commonly prescribed oral antidiabetic medicine prescribed 189 (90.0%). Concerning the presence of comorbidity, 104 (49.5%) patients reported they had a comorbid illness. Hypertension is the most common comorbidity 45 (43.3%) followed by Erectile dysfunction 32 (30.7%) and chronic kidney disease 24 (23.1%). One hundred thirty‐two (62.9%) patients reported that they had complications secondary to diabetes. Concerning lifestyle factors, the majority 166 (79.0%) were non‐smokers. About one‐half 108 (51.4%) of the patients were physically active. Among the physically active patients, 76 (70.4%) performed physical activity to the recommended level (Table 2). Regarding foods recommended, patients had good awareness about cholesterol‐free substitutes 58 (27.6%) followed by 49 (23.3%) and vegetables and fruits 52 (24.8%). Concerning foods to avoid, 91 (43.3%), 57 (27.1%) and 49 (23.3%) reported that whole egg and whole milk, regular meat, and saturated oils and butter should be avoided from a diabetic diet (Figure 2). Concerning medication adherence, 51 (24.3%) of patients were adherent to prescribed medications (Table 3).

**TABLE 2:** Disease related and life‐style factors of adult type 2 diabetics at Gamo Gofa Zone, Southern Ethiopia

Variables		Frequency	Percent
Duration of disease	Below 5 years	113	53.8
	5 years and above	97	46.2
Type of medication	Oral antidiabetics	179	85.2
	Insulin and oral antidiabetics	31	14.8
Family history of diabetes	None	147	70.0
	1st relative	50	23.8
	2nd relative	13	6.2
Fasting blood glucose	80–130 mg/dl	63	30.0
	Above 130 mg/dl	147	70.0
MBI in Kg/m^2^	18–24.9 kg/m^2^	117	55.7
	25–30 kg/m^2^	87	41.4
	Above 30 kg/m^2^	6	2.9
Presence of comorbidity	Yes	104	49.5
	No	106	50.5
Type of comorbidity (*n* = 104)	Hypertension	45	43.3
	Heart failure	3	4.8
	Kidney disease	24	23.1
	Erectile dysfunction	32	30.7
Presence of complications	Yes	132	62.9
	No	78	37.1
Type of Complications of Diabetes (*n* = 132)	Hyperglycaemia related complications	105	79.5
	Diabetic neuropathy	13	9.8
	Diabetic retinopathy	11	8.4
	Foot ulcer	3	2.3
Smoking status	Never	166	79.0
	Ex‐smoker	44	21.0
Physical activity	Yes	108	51.4
	No	102	48.6
Frequency of physical activity (*n* = 108)	Daily	16	7.6
	Every other day	17	8.1
	Three times a week	43	20.5
	Once a week	32	15.2
Dietary advice and meal schedule by nurse (*n* = 210)	Yes	189	90.0
	No	21	10.0
Frequency of meal plan followed	Never	27	12.9
	Sometimes	135	64.3
	Always	48	22.9

**TABLE 3:** Medication adherence status based on Morisky Medication Adherence Scale‐8 (MMAS‐8) among adult type 2 diabetics at Gamo Gofa Zone

Morisky Medication Adherence Scale‐ MMAS‐8		Frequency (%)
Do you sometimes forget to take your diabetes medication	Yes	91 (43.3%)
	No	119 (56.7%)
In the last 2 weeks, was there any day when you did not take your diabetes medication	Yes	56 (26.7%)
	No	154 (73.3%)
Stopped or decreased dose for any reason	Yes	13 (6.2%)
	No	197 (93.8%)
Forgot during travelling	Yes	80 (38.1%)
	No	130 (61.9%)
Did you take your medication yesterday	Yes	200 (95.2%)
	No	10 (4.8%)
Have you sometimes Stop taking medication when feeling better	Yes	24 (11.4%)
	No	186 (88.6%)
Have you ever felt distressed for strictly following your diabetes treatment	Yes	70 (33.3%)
	No	140 (66.7%)
How often do you have difficulty to remember taking all your diabetes medications	Never almost never	98 (46.7%)
	Sometimes	91 (43.3%)
	Frequently	21(10.0%)
Overall adherence	Adherent (0–2 score)	51 (24.3%)
	Non‐adherent (≥3)	159 (75.7%)

### 3.3 Diabetes knowledge and attitude

**FIGURE 3:**
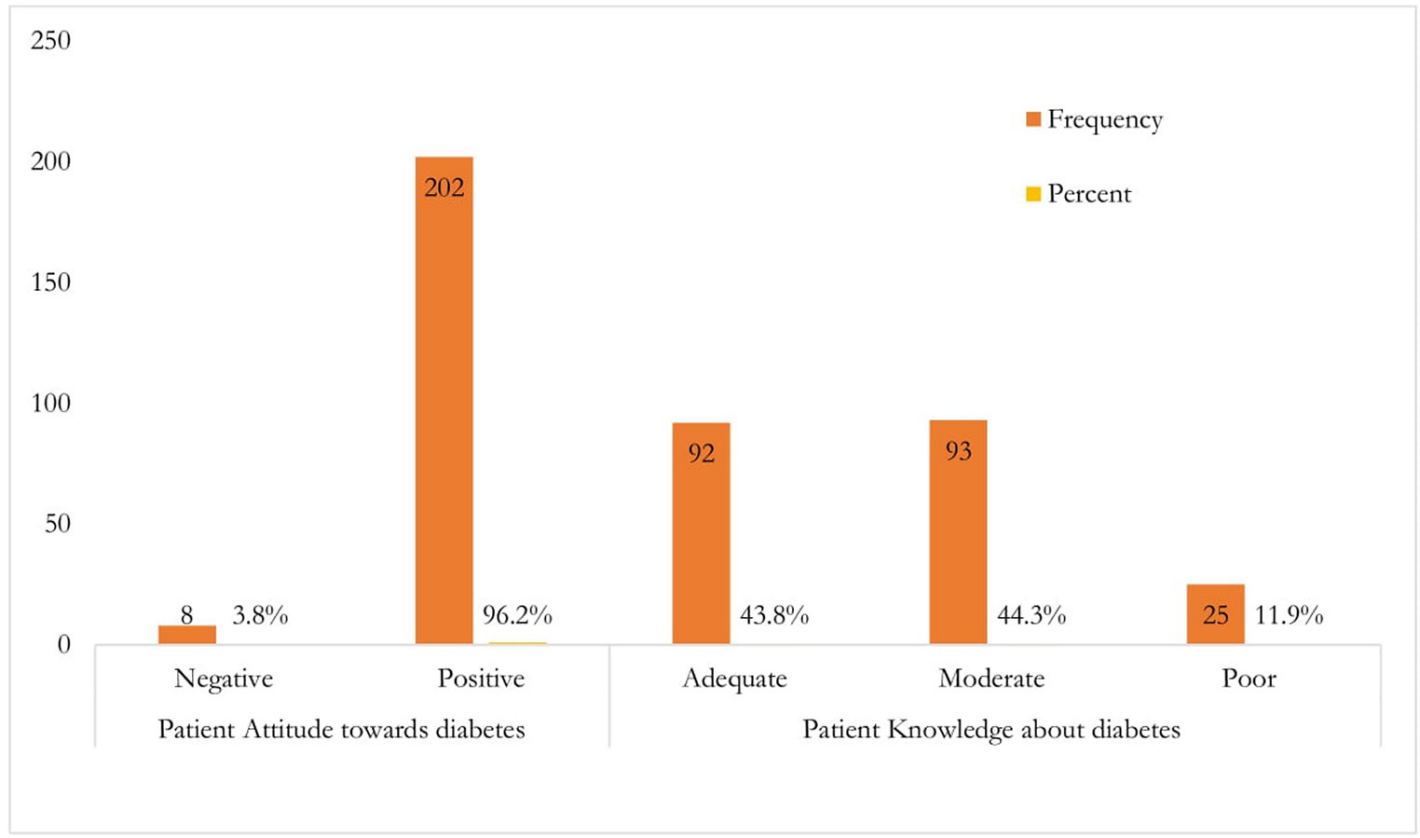
Patient diabetes attitude and knowledge among adult type 2 diabetics at Gamo and Gofa zones, Southern Ethiopia

Concerning diabetes attitude, the mean score of positive attitude questions on the Likert scale of five was 13.89 ± 3.74 ranging from 6 to 22 and the mean score of negative attitude questions was 16.11 ± 3.43 ranging from 5 to 21. The overall attitude mean score was 37.45 ± 4.52 ranging from 25 to 45. The majority of patients 202 (96.2%) had a positive attitude towards diabetes followed by a negative attitude 8 (3.8%). Concerning diabetes knowledge, 25 (12%) of patients had poor knowledge concerning diabetes and its care process. Ninety‐three (44.3%) patients had moderate knowledge and 92 (44%) had adequate knowledge about diabetes and its care process (Figure 3).

### 3.4 Structural aspects of quality care

A quality improvement system and an operational plan for diabetes management are available in all included facilities. However, diabetes‐specific evidence‐based guidelines, operational plans to reduce overweight and obesity, and operational plan to reduce physical inactivity were not available in all three included facilities. Concerning the availability of basic technologies, the glycated haemoglobin concentration (HbA1c %) test and foot vascular status by Doppler test were not available. Concerning general availability of diabetes medications (i.e., greater or equal to 50% availability), all facilities have oral antidiabetic medicines. None of the facilities had retinal photocoagulation and renal replacement therapy. All facilities had unique medical record numbers assigned to each patient. However, only one facility had a computerized diabetic‐specific registry. Only one hospital performed medical record auditing, data quality checks, archiving procedures and takes corrective actions regularly. Similarly, only one hospital had an automated health information system through the implementation of an integrated electronic medical record system (Table 4).

**TABLE 4:** Status health facility structure for providing quality diabetes care at Gamo Gofa zone, Southern Ethiopia

Structure related factors	Met (1)	Unmet (0)
1. Availability Quality improvement system (interview head of facility)	1	0
2. Availability of policy documents		
2.1. Operational plan for diabetes management	1	0
2.2. Evidence based diabetes specific guidelines Available in your facility?	0	1
2.3. Operational plan to reduce overweight and obesity	0	1
2.4. Operational plan to reduce physical inactivity:	0	1
3. Availability of basic technologies for diabetes:		
A. Oral glucose tolerance test	0	1
B. HbA1c test	0	1
C. Foot vibration perception by tuning fork	1	
D. Foot vascular status by Doppler	0	1
E. Urine strips for glucose and ketone measurement	1	0
4. General Availability of diabetes medications (i.e., ≥50% availability)	1	0
5. Availability of diabetes registry	1/3	0
6. Retinal photocoagulation	0	1
7. Renal replacement therapy	0	1
8. Medical record management	Met	Unmet
8.1. Unique medical record number is assigned to a patient during his/her first visit of care.	1	0
8.2. The hospital performs medical record auditing, data quality checks, archiving/culling procedures and takes corrective actions on a regular basis.	1/3	2/3
8.3. The hospital ensures patient's medical records return from different service units to medical records unit at the end of each service day in accordance with medical record tracing system.	1	0
8.4. The hospital automated health information system through implementation of integrated electronic medical record system.	1/3	2/3

### 3.5 Process and outcome indicators for type 2 diabetes

In this study, we operationalized process and outcome indicators as the presence of regular FBG and HbA1c% monitoring, measurement of BP in each visit, lipid profile testing based on the low‐density lipoprotein (LDL) cholesterol at least annually, measuring patient BMI and providing obesity reduction measures, renal function test for all patients during initiation of therapy particularly those starting with metformin based regimen, screening for the presence of diabetic complications (neuropathy and retinopathy), presence of smoking cessation interventions and improving patient satisfaction and reducing diabetes‐related distress and their respective targets.^40^ There was no lipid profile testing, no renal function testing and no glycated haemoglobin A1C% (HbA1C %) testing. Only 63 (30%) patients achieved their recommended FBG level. Blood pressure level was measured regularly and recorded only for 104 (49.5%) patients. The mean systolic BP was 123.36 ± 11.07 ranging from 100 to 150 mmHg. Mean diastolic BP was 79.2 ± 6.5 mmHg ranging from 68 to 90 mmHg. Concerning treatment outcomes (FBG, BP and BMI), only 41 (19.5%) achieved the recommended target value for the three variables (Table 5).

**TABLE 5:** Process and outcome indicators for quality of diabetes care

Process and outcome indicators for type 2 diabetes		Frequency	Percent
Average Fasting blood sugar in (mg/dl)	80–130 mg/dl	63	30.0
	>130 mg/dl	147	70.0
Frequency of blood glucose monitoring	Monthly	143	68.1
	Quarterly	12	5.7
	Twice Annually	3	1.4
	Every visit	52	24.8
Treatment given initially	Metformin	107	51.0
	Insulin + oral antidiabetics	31	14.7
	Metformin + Glibenclamide	72	34.3
Metformin dose (*n* = 179)	500 mg daily	37	17.6
	500 mg bid	124	59.0
	1gm morning and 500 mg evening	14	6.7
	1 g bid	4	1.9
History of hospitalization	Not hospitalized	6	2.9
	Hospitalized	204	97.1
Documented reason for hospitalization (*n* = 204)	Diabetes as primary diagnosis	97	47.5
	Cardiovascular diseases	59	28.9
	Infections	43	21.1
	Asthma	5	2.5
BP category (*n* = 104)	<120/80 mmHg	10	9.6
	120–129/80–89 mmHg	49	47.1
	130–139/80–89 mmHg	15	14.4
	≥140/90 mmHg	30	28.8
Taking antihypertensive treatment (*n* = 96)	Yes	45	43.3
	No	59	56.7
Antihypertensive treatment given (n = 45)	Hydrochlorothiazide	9	20.0
	Enalapril	13	28.8
	Hydrochlorothiazide + Enalapril/captopril	23	51.1
Intermediate composite outcome (FBG, BP and BMI control)	Yes	41	19.5
	No	169	80.5

### 3.6 Screening diabetes complications

Concerning ophthalmologist screening for diabetes‐associated retinopathy, 94 (44.8%) of patients were referred to ophthalmologist consultation. Regarding neuropathy screening based on MNSI, the mean score of neuropathy screening was 4.48 ± 2.24 ranging from 2 to 13 for the patient‐reported part. Thirty‐four (16.2%) of patients had a mean score of ≥7 indicating the presence of neuropathy and the need for referral to further evaluation. Similarly, the mean score of neuropathy assessment by clinicians was 1.033 ± 1.41 ranging from zero to four. Thirty‐five (16.6%) patients had a physical assessment mean score greater than or equal to three. A total of 67 (31.9%) of patients could benefit from neuropathy screening and referral (Table 6).

**TABLE 6:** Measure of distal symmetrical peripheral neuropathy among adult type 2 diabetics at selected public hospitals in Gamo Gofa Zone, Southern Ethiopia

		Frequency	Percent
Ophthalmologic screening			
Visited ophthalmology for eye check (*n* = 210)	Yes	94	44.8
	No	116	55.2
Frequency of ophthalmology visit (*n* = 94)	Monthly	6	2.9
	Quarterly	5	2.4
	Twice yearly	32	15.2
	Yearly	51	24.3
Reasons for not visiting ophthalmology clinic (*n* = 116)	No eye clinic	5	4.3
	My doctor did not tell me	73	62.9
	Lack of money	33	28.5
	Others	5	4.3
Michigan Neuropathy Screening Instrument for distal symmetrical neuropathy			
A. Completed by the person with diabetes	Mean score out of 15		
	<7	176	83.8
	≥7	34	16.2
Physical Assessment (completed by health professional)	Mean score out of 10		
	<3	175	83.3
	≥3	35	16.7
	Total	210	100.0

### 3.7 Patient satisfaction

The overall satisfaction score was 16.6 out of 35 points, which was below the mean value. Overall, patients were not satisfied by the type 2 diabetes care. Patients were mainly dissatisfied with the technical quality of care (1.81 out of five) followed by interpersonal manner (2.16 out of 5). The overall distress is not worthy of clinical attention (mean of respective item score was <3) (Table 7).

**TABLE 7:** Summary of patient satisfaction on type 2 Diabetes care and diabetes related distress at selected public hospitals in Gamo Gofa Zone, Southern Ethiopia

S. No	Patient satisfaction on type 2 Diabetes care	Sub‐item sum	Items in category	Satisfaction score
1	General satisfaction score, (Q3+ reverse Q17 score)/2	7.1	2	3.5
2	Technical quality, (Q4 + Q14 + reverse Q6)/3	5.43	3	1.81
3	Interpersonal manner, (Q10 + reverse Q11)/2	4.32	2	2.16
4	Communication, (Q1 + reverse Q13)/2	7.20	2	3.60
5	Financial aspects, (Q5 + reverse Q7)/2	5.89	2	2.95
6	Time spent with Doctor, (Q15 + reverse Q12)/2	5.88	2	2.94
7	Accessibility and convenience, (Q8 + Q18 + reverse Q9+ reverse Q16)/4	12.55	4	3.14
	Overall satisfaction		17	16.6

S. No	In General I am feeling	Sub‐item sum	Number of items in sub‐group	Respective mean value
1	Emotional burden	9.3	5	1.86
2	Physician‐related Distress	6.44	4	1.6
3	Regimen‐related Distress	8.41	5	1.68
4	Interpersonal Distress	4.6	3	1.5
5	Overall diabetes distress	28.75	17	1.69

### 3.8 Overall quality of diabetes care

Overall quality of diabetes care, for structure mean score [6.7/12 = 0.56 (i.e., quality improvement system = 2, availability of policy documents = 0.25, availability of basic technologies =0.8, general availability of essential medicines = 2, availability of diabetes registry = 0.33, photocoagulation = 0, renal replacement therapy = 0, medical document management = 1.33)]; process (10/16 = 0.625) [i.e., no HbA1C%, LDL test, ophthalmology screening, renal function test). For outcome (5.7/16 = 0.357) [FBG = 30% = 0.6, BMI = 55.7% = 1.114, smoking cessation = 100% = 2, neuropathy screening = 100% = 2, distress management = 100% = 2]. Therefore, score of all three sub‐components of quality care was below the agreed minimum score, and quality of care provided to adult type 2 diabetes was poor.

## 4 DISCUSSION

### 4.1 General description of the study

In this study, we assessed the quality care provided to 210 adults with type 2 diabetes at public hospitals in Gamo, Gofa Zone, based on the structure‐process‐outcome (SPO) triad. A majority 179 (85.2%) of patients were taking oral antidiabetic medications. Metformin was the most commonly used oral‐antidiabetic alone or in combination 189 (90.0%). This is supported by the evidence from recent recommendations that suggest metformin as the preferred initial pharmacologic agent for the treatment of type 2 diabetes.^20^ However, metformin dose intensification was done only for 18 (10.1%) patients. However, the metformin dose should be escalated to get maximum cardiac and blood glucose control benefits in the specified period since the suggested minimum effective daily dose of metformin is 1500 mg/day.^41^ This sub‐optimal dosing of metformin could be due to the fear of metformin‐associated side‐effects since there was no periodic testing for renal function.

One hundred four (49.5%) patients reported they had a comorbid illness. Hypertension was the most common comorbidity 45 (43.3%) followed by erectile dysfunction 32 (30.7%) and chronic kidney disease 24 (23.1%). A retrospective database study from Germany also showed hypertension (66.5%) and obesity (18.7%) as the most commonly diagnosed comorbidities.^42^ Hypertension and diabetes share a significant overlap in underlying risk factors including (ethnicity, familial, dyslipidaemia and lifestyle determinants) and complications.^43^ Patients with diabetes are more likely to develop hypertension with the incidence of hypertension being twofold higher in those with diabetes relative to similarly aged individuals without diabetes.^44^

One hundred thirty‐two (62.9%) patients reported that they had complications secondary to diabetes. Hyperglycaemia‐associated complications were the most commonly reported complications followed by diabetic neuropathy 68 (32.4%). This is supported by evidence from a cross‐sectional study conducted to determine the prevalence and reasons for hospitalization in adults with diabetes in Kuwait showed that diabetes was the principal or secondary diagnosis in 40.6% of hospitalizations. Unrecognized diabetes or new hyperglycaemia was found in 12.9% of the patients.^45^

The majority of 195 (88%) of patients had adequate knowledge about diabetes and its care process. Diabetes knowledge was not associated with composite intermediate outcomes. This is in line with evidence from a cross‐sectional study conducted to explore the association between knowledge on diabetes and glycaemic control among patients with type 2 diabetes in Bangladesh showed that 45.6% of participants had good, 37.7% moderate and 16.7% poor knowledge on diabetes. Knowledge about diabetes was not associated with glycated haemoglobin (HbA1c).^46^

### 4.2 Quality of diabetes care

All three sub‐components of quality care (SPO) were below the agreed minimum score, and the quality of care provided to adult type 2 diabetes was poor. This was also implicated in patients' dissatisfaction with the technical quality of care provided to them. Therefore, it is important to address the entire diabetes care system at the facility level could improve the quality of care and patient satisfaction. The quality of diabetes care can be influenced by the healthcare structure (lack of evidence‐based guidelines; poor team involvement diabetes management; poor medication adherence tracking at a system level; poor implementation of electronic health records; poor patient education about diabetes and its care process including healthy lifestyles).^15^, ^47^, ^48^, ^49^, ^50^ To improve the quality of diabetes care, one should address and revitalize delivery system design, self‐management support, decision support, clinical information systems, community resources and policies and health systems.

Concerning the process indicators, there is no glycated haemoglobin A1C% (HbA1c %) testing, no lipid profile test recorded, no renal function test recorded and inadequate ophthalmologist screening for retinopathy. This is supported by evidence from a health facility‐based cross‐sectional study conducted in the Jimma zone that indicated the FBG test was conducted for 85.6% of the cases and none of the patients received the HbA1c test.^15^, ^16^ However, the NDQIA set quality indicators to included process measures (HbA1c% tests, at least one lipid profile, any test for microalbuminuria, dilated retinal eye and foot examination, influenza immunization, aspirin use, smoking cessation and pregnancy counselling).^51^, ^52^, ^53^ A retrospective cohort study conducted to evaluate the quality of diabetes care in Egypt showed that annual testing for total cholesterol, triglycerides and albuminuria was 60.6%, 52.6% and 10.3%, respectively.^54^ This difference could be explained by the difference in the level of the healthcare system and availability and affordability of HbA1c%, serum creatinine and lipid profile tests.

It is recommended to screen type 2 diabetes patients for *Retinopathy* (screen the retina every 1–2 years using the best available test, preferably a non‐mydriatic retinal photography), *Nephropathy* [screen for albumin in urine every year, and measuring serum creatinine every year to calculate eGFR once albuminuria is detected and/or when other risk factors are present (e.g., hypertension)], *Peripheral neuropathy* (using the 5.07 monofilament to identify if the foot is at risk and inspecting the feet at every visit when they are at risk and educate the patient on prevention of diabetic foot), and *Macrovascular diseases* when the patient has typical or atypical symptoms (screen for peripheral artery disease by palpating the foot pulses and/or measuring the SBP to calculate the ankle/brachial index).^40^ Concerning screening for diabetes complications, diabetes eye and neuropathy examinations were done for 94 (44.8%) and 210 (100%) patients, respectively. This is higher than findings from a study conducted in Jimma university specialized hospital showed that diabetes eye and neurologic evaluations were ever done for 42.9% and 9.4% of patients, respectively.^49^ This difference could be explained by the difference in the study period and associated improvement in the health care system.

There is persistent variability in the quality of diabetes care across providers.^14^ Most of guidelines agree on HbA1c%, LDL, and BP as type 2 diabetes quality care outcome.^11^, ^12^ Maintaining an A1C level of about 7%, keeping blood pressure <140/90 mmHg and maintaining LDL at <100 mg/dl (with no cardiovascular disease) and an LDL of <70 mg/dl with any type of cardiovascular complications are key proponents of diabetes management.^55^ Our intention was to evaluate the above agreed outcomes (HbA1C%, BP and LDL‐cholesterol) control. However, there was no HbA1c%, LDL‐cholesterol, microalbuminuria test report, we used surrogate outcome indicators (FBG, BP and BMI) and only 41 (19.5%) achieved the recommended target value for the three variables. This is lower than the findings from eight European countries,^56^ and findings from an assessment of the quality of care given to diabetic patients at Jimma University Specialized Hospital showed that 26.9% of patients had mean FBS levels below 130 mg/dl.^49^ Blood pressure level was measured regularly and recorded only for 104 (49.5%) of patients and 30 (28.8%) patients had hypertension and 15 (14.4%) had stage one hypertension (130–139/80–89 mmHg).^49^ The difference could be explained by the level of the healthcare system, socioeconomic status of patients.

### 4.3 Strengths and limitations

The strength of this study relies on its methodology (i.e., using validated quality of care assessment model adapted to the country context, using composite intermediate outcome and using primary data source). However, the findings of this study should be applied in light of its limitations. Quality of care is also affected by provider‐oriented factors. We did not include the provider‐oriented factors such as lack of knowledge about guidelines, number of professionals and training of practice team. In addition to this, long‐term outcomes such as mortality and the health status of the population were not evaluated.

## 5 CONCLUSION

The overall quality of care provided to adult type 2 diabetes patients was poor, particularly in areas such as the availability of evidence‐based guidelines, operational plan to reduce obesity, monitoring of lipid profile and glycaemic control. Therefore, developing strategies for addressing structure, process and outcome‐related gaps by involving all stakeholders (patients, providers and health systems) is critical to improving the quality of care provided to adults with type 2 diabetes. Futures studies with better methodological quality and involving provider‐related factors and long‐term outcomes on wider population with type 2 diabetes are important to determine the impact of quality of care on diabetes outcomes.

## AUTHOR CONTRIBUTIONS

**Teklu Teshome Russo:** Formal analysis (equal); writing – original draft (equal). **Mende Mensa Sorato:** Conceptualization (equal); data curation (equal); formal analysis (lead); methodology (lead); validation (lead); writing – original draft (lead); writing – review and editing (lead). **Akililu Ayele Mesfin:** Data curation (equal); formal analysis (equal). **Tadiwos Hailu:** Writing – review and editing (equal). **Abayneh Tunje Tanga:** Writing – review and editing (equal). **Zebenay Bussa:** Writing – review and editing (equal).

## CONFLICT OF INTEREST

The authors declare that they have no competing interests.

## ETHICS APPROVAL AND CONSENT TO PARTICIPATE

Ethical clearance was obtained from institutional review board of Arba Minch University College of medicine and health sciences, with project code number: GOV/AMU/TH14/CMHS/SoM/05/10. Permission letters to conduct the study was obtained from respective hospital administrations. Interview was carried out only with full consent of the patient being interviewed. Each respondent was assured about confidentiality of information provided by them.

## CONSENT FOR PUBLICATION

All authors read the full version of this manuscript and agreed to publish.

## Data Availability

All the data reported in the manuscript are publicly available upon acceptance of the manuscript.
